# Structural basis of Sphingosine-1-phosphate transport via human SPNS2

**DOI:** 10.1038/s41422-023-00913-0

**Published:** 2023-12-20

**Authors:** Yaning Duan, Nancy C. P. Leong, Jing Zhao, Yu Zhang, Dat T. Nguyen, Hoa T. T. Ha, Na Wang, Ruixue Xia, Zhenmei Xu, Zhengxiong Ma, Yu Qian, Han Yin, Xinyan Zhu, Anqi Zhang, Changyou Guo, Yu Xia, Long N. Nguyen, Yuanzheng He

**Affiliations:** 1 Laboratory of Receptor Structure and Signaling, HIT Center for Life Sciences, School of Life Science and Technology, Harbin Institute of Technology, Harbin, Heilongjiang China; 2 Department of Biochemistry, Yong Loo Lin School of Medicine, National University of Singapore, Singapore, Singapore; 3 MOE Key Laboratory of Bioorganic Phosphorus Chemistry & Chemical Biology, Department of Chemistry, Tsinghua University, Beijing, China; 4 Life Sciences Institute, Immunology Programme, National University of Singapore, Singapore, Singapore; 5 Immunology Translational Research Program, Yong Loo Lin School of Medicine, National University of Singapore, Singapore, Singapore; 6 Singapore Lipidomics Incubator (SLING), Life Sciences Institute, National University of Singapore, Singapore, Singapore

**Keywords:** Cryoelectron microscopy, Transport carrier

Dear Editor,

Sphingosine 1-phosphate (S1P) plays crucial roles in various physiological processes, including immune response, vascular development, and neural system homeostasis^1^ through activating S1P receptors (S1PRs). Notably, a desirable concentration gradient of S1P which is achieved by S1P transporters, including spinster homolog 2 protein (SPNS2) and MFSD2B that transport S1P from the intracellular side to the extracellular side of cells,^2,3^ is essential for proper S1P signaling. SPNS2 and MFSD2B belong to the major facilitator superfamily (MFS), and specifically, SPNS2 belongs to the endosomal spinster subfamily.^4^ S1PR modulators, such as Fingolimod (FTY720), have been approved for treating multiple sclerosis.^1^ Nevertheless, S1PR modulators often exert undesirable effects on the patients, highlighting the need for a better target. Recent findings suggested that targeting SPNS2 has shown promise in reducing disease severity of autoimmune diseases.^5,6^ Together with the fact that the reported lysolipid transporter MFSD2A^7,8^ and a bacterial homolog of spinster *Hyphomonas neptunium* (*Hn*SPNS) structures^9^ can only offer limited insights into the S1P transport mechanism due to their low sequence homology (Supplementary information, Fig. S1), suggesting an imminent need for the structural determination of SPNS2.

We expressed full-length human SPNS2 in *Spodoptera frugiperda* (*Sf9*) insect cells and purified the protein (Supplementary information, Fig. S2a). To overcome the difficulty of small-size membrane protein in cryo-electron microscopy (cryo-EM) structural solving, we developed a nanobody against SPNS2. Together with the NabFab^10^ and the anti-Fab Nb, we were able to solve SPNS2 in apo, S1P-bound, FTY720-P-bound, and inhibitor 16d-bound states at resolutions of 3.29 Å, 3.22 Å, 2.98 Å, and 3.18 Å, respectively, by the gold standard of FSC = 0.143 (Fig. 1a; Supplementary information, Figs. S2b, S3, S4a and Table S1). The bindings of S1P (18:1) and FTY720-P to SPNS2 were confirmed by the liquid chromatography-mass spectrometry (LC-MS) analysis of purified SPNS2 complex (Supplementary information, Fig. S2c).

**Fig. 1:**
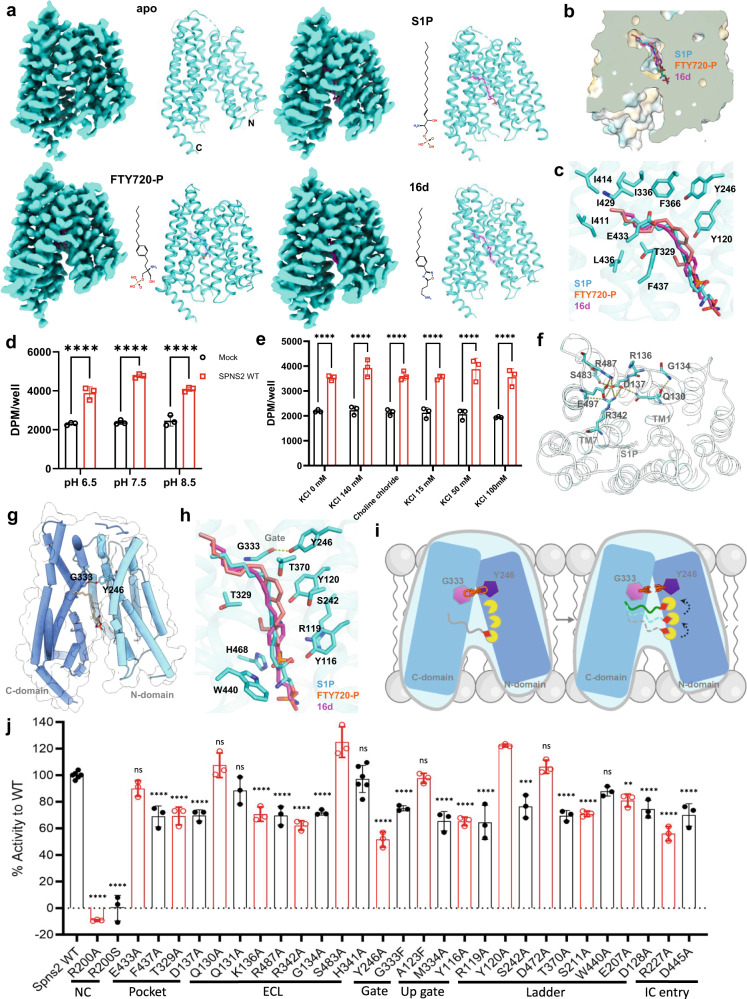
Structural basis of S1P transport via human SPNS2. **a** Overall structures of SPNS2 in apo, S1P-bound, FTY720-P-bound and 16d-bound states. Left panel, orthogonal views of the cryo-EM density map; right panel, a model of the complex in the same view. **b** The hydrophobic pocket for the lipophilic tail of S1P and FTY720-P. The yellow surface color indicates a hydrophobic region, and the blue color indicates a hydrophilic region. **c** The detail of the hydrophobic pocket for substrate binding. **d** The effect of changing extracellular pH on the transport activity of SPNS2. **e** The effect of changing extracellular potassium and sodium concentration on the transport activity of SPNS2. **f** A polar interaction network on the extracellular side of SPNS2. **g** A gate formed by the hydrogen bond interaction between Y246 and G333 to lock SPNS2 in an inward-facing state. **h** A hydrophilic tunnel filled with polar residues below the G333/Y246 gate. **i** A proposed “ladder” mechanism for S1P transport via SPNS2. **j** The transport activities of SPNS2 mutants. Data are presented as mean ± SD; *n* = 3 independent samples; n.s., no significance; *P < 0.05; ** *P* < 0.01; ***P < 0.001, ****P < 0.0001. One-way and Two-way ANOVA were used.

The overall structure of SPNS2 shows a classical MFS^4^ 12 transmembrane (TM) helices folded into two pseudosymmetries of six-helix bundles, namely, the N-domain (TM1–6) and C-domain (TM7–12) (Supplementary information, Fig. S4b). The cryo-EM structure of SPNS2 adopted an inward-facing conformation (Fig. 1a). The structure is similar to the AlphaFold prediction of SPNS2 with a root mean square deviation (RMSD) of 1.137 Å over 406 Cα (Supplementary information, Fig. S4c). The N- and C-domains are held together on the extracellular side via 3 layers of helices interactions and cross-layer interactions (Supplementary information, Fig. S4d). A comparison of apo, S1P-bound, FTY720-P-bound, and 16d-bound shows that these SPNS2 structures are nearly identical (RMSD of 0.478 Å over 423 pairs of Cα) (Supplementary information, Fig. S4e).

We developed an S1P transport assay to assess the transport activity of SPNS2. Radioactive [^3^H] sphingosine was incubated with HEK293 cells co-transfected with SPNS2 and sphingosine kinase 2 to synthesize S1P for 3 h. Then the cells were incubated with DMEM containing 0.5% BSA to stimulate S1P release into the medium. Both cell medium and cell pellets were collected for S1P isolation and quantitation. We employed untagged SPNS2 and mutant constructs because tagging GFP to the C-terminus affects SPNS2 localization and transport activity (Supplementary information, Fig. S4f). We confirmed that the radioactive signal released out in the SPNS2 expressing cells was S1P using the LC-MS/MS approach (Supplementary information, Fig. S5a). Furthermore, using proteoliposomes containing SPNS2, we showed that SPNS2 directly transports S1P, but not sphingosine (Supplementary information Fig. S5b–g). In the wild-type SPNS2, we observed an increase in the amount of S1P in the supernatant, accompanied by a decrease of S1P in the cell pellet. In contrast, the reported inactive mutant R200A^2^ showed minimal changes in S1P levels between the supernatant and the cell pellets (Supplementary information, Fig. S4f). We utilized this method to address several key questions regarding SPNS2-mediated S1P transport mechanisms: whether the transport activity is driven by a gradient of cations across the cell membrane and what are the key amino acids for cargo transporting. The results suggest that SPNS2 is not a proton or sodium/potassium-dependent transporter (Fig. 1d, e).

Our structures showed that the lipophilic carbon chain of S1P, FTY720-P, and 16d is inserted into a hydrophobic pocket formed by residues from TM7, 8, 9, 10 and 12, while the charged headgroup is positioned in the center of the intracellular cavity. The hydrophobic pocket is composed of residues I411^TM9^ (superscripts refer the localization of the residue), I414^TM9^, I429^TM10^, L436^TM10^, L332^TM7^, I336^TM7^, F366^TM8^, L436^TM10^ and F437^TM10^ (Fig. 1b, c). We also spotted several polar residues such as T329 ^TM7^, E433 ^TM10^ and Y120 ^TM1^ at the entry of the hydrophobic cavity. The densities of the carbon chains are of good quality, while the densities of the polar headgroup are relatively weak (Fig. 1a; Supplementary information, Fig. S3c), which may be due to the lack of restraint on the headgroup within a large empty cavity.

A structural comparison of the chicken MFSD2A^7^ with S1P-bound SPNS2 shows that SPNS2 largely differs from MFSD2A (RMSD = 4.16 Å over 328 pairs of Cα) on the extracellular loop 3 (ECL3) between TM5 and TM6 (Supplementary information, Fig. S6a). In the substrate binding site, both S1P and lysophosphatidylcholine (LPC) utilize a similar hydrophobic pocket for tail engagement. However, the orientation of the substrate headgroups is markedly different. LPC points towards the lateral entry between TM5 and TM8, while S1P points towards the center of the cavity, close to the TM2 and TM11 sides. In contrast to the generally hydrophilic cavity of SPNS2, the cavity on the intracellular side of MFSD2A exhibits a hydrophobic nature at the C-domain and a hydrophilic nature at the N-domain (Supplementary information, Fig, S6b). We also compared SPNS2 structure with the bacterial *Hn*SPNS^9^ (RMSD = 3.492 Å over 296 Cα), which shows that the most notable feature is the two extended blobs on the extracellular side of *Hn*SPNS (Supplementary information, Fig. S6c).

An electrostatic analysis of the extracellular side of SPNS2 reveals that the N-domain is negatively charged and the C-domain is positively charged (Supplementary information, Fig. S6d). Interestingly, there is a patch of negatively charged area near the border between the N- and C-domains. A closer examination of this region reveals an extensive network of polar interactions that tightly holds the N-/C-domain together. Specifically, Q130^TM1^, D137^TM2^, R342^TM7^ and S483^TM11^ form the core network, and E497^TM12^, R487^TM11^, R136^TM2^ and G134^ECL1^ form the peripheral network (Fig. 1f). Mutagenesis study shows that the mutants G134A and D137A have substantially decreased transport activity of SPNS2 (Fig. 1j; Supplementary information, Fig. S6f). On the entry of the intracellular side, D445^TM10^ forms an ionic interaction with R389^TM8^ and R227^TM5^ forms a hydrogen-bond interaction with the backbone carbonyl groups of F222^ICL2^ and G219^TM4^ (Supplementary information, Fig. S6e). Mutants of R227A and D445 substantially decrease the transport activity of SPNS2 (Fig. 1j; Supplementary information, Fig. S6f).

The opening and closing of the gating region is the central mechanism of cargo transport. In SPNS2, the gating region is primarily formed by the interactions from TM1/TM7 of Layer II (Supplementary information, Fig. S4d), along with the cross-layer interactions involving TM5 and TM2. One hydrogen bond interaction, formed by the hydroxyl group of the tyrosine ring of Y246^TM5^ and the carbonyl group of the backbone of G333^TM7^, is of particular interest (Fig. 1g). Molecular dynamics (MD) simulations of apo SPNS2 show that the hydrogen bond is very stable during the simulations (Supplementary information, Fig. S7a). Additionally, hydrophobic residue M334 sits above the gate, guarding the unprepared entry of the polar headgroup of the cargo (Supplementary information, Fig. S7d). Mutants of Y246A, G333F and M334A had substantially decreased transport activity of SPNS2 (Fig. 1j; Supplementary information, Fig. S6f).

The gate area is connected to the intracellular cavity by an inverted funnel filled with polar residues, including T329^TM7^, H468^TM11^, R119^TM1^, Y120^TM1^, Y246^TM5^, Y116^TM1^, S242^TM5^ and T370^TM8^. These residues form multiple layers of polar “ladder” around the cavity (Fig. 1h), which may provide multiple anchoring points for the phosphate group of a substrate to engage with the transporter during the transportation. Mutations of these polar residues substantially decreased the transport activity of SPNS2 (Fig. 1j; Supplementary information, Fig. S6f), suggesting that the phosphate head of substrate may utilize the polar residues, such as R119^TM1^, Y120^TM1^ and Y246^TM5^, as ladders, to climb up to the gate during cargo transport (Fig. 1i). Interestingly, in the extended MD simulations of FTY720-P-bound SPNS2 (1 µs), we observed that the phosphate head of the lipid climbs up and forms polar interactions with the ladder residues at the wall of the intracellular cavity (Supplementary information, Fig. S7b, c). This observation aligns well with recent structures of zebrafish MFSD2A in different states.^11^

We also compared the key polar residues forming the “ladder” of SPNS2 with MFSD2A.^7^ The comparison shows that the polar “ladder” is not conserved in MFSD2A (Supplementary information, Fig. S7e). The change of polar residues to hydrophobic residues in MFSD2A is consistent with the releasing function of MFSD2A on the intracellular side where the cargo (e.g., LPC) is released. This may explain why SPNS2 and MFSD2A transport lysolipids in reverse directions: a highly polarized cavity of SPNS2 (Fig. 1h) facilitates lysolipid loading on the intracellular side, while a more hydrophobic and neutral cavity of MFSD2A (Supplementary information, Fig. S6b) promotes the releasing and exit of the substrate on the intracellular side. Despite the difference in transport direction, the general lipid transport mechanism of SPNS2 closely resembles the “rock-and-swing” mechanism observed in MFSD2A.^12^ Notably, a recent investigation of a zebrafish counterpart of MFSD2A has proposed a lipid-flipping mechanism^11^ that bears a remarkable resemblance to the “climbing ladder” phenomenon we observed in our MD simulations (Supplementary information, Fig. S7b, c). We further compared the “ladder” of SPNS2 with that of *Hn*SPNS.^9^ In *Hn*SPNS, the large polar residues Y246^TM5^ and Y120^TM1^ are replaced by Q168 and T143, respectively (Supplementary information, Fig. S7f). Additionally, we examined the position for the loss of function mutant *ko157* (R200S of human) of zebrafish.^2^ R200^TM4^ is in close contact with R119^TM1^ which serves as a step in the ladder for the phosphate group of substrates to reach the gating area. Besides, the charged side chain of R200^TM4^ may also form a cation–π interaction with F147^TM2^ which is in close contact with S1P (Supplementary information, Fig. S7g).

The recent identification of SPNS2 inhibitors^5^ highlights the potential of developing these inhibitors for the treatment of autoimmune disorders. Unlike the substrates of SPNS2, which have a negatively charged phosphate group (Fig. 1a), the head of inhibitor 16d is positively charged. We found that the two positively charged residues R119 and H468 of SPNS2 sit above the positively charged head of 16d (Supplementary information, Fig. S7h, i) in the cryo-EM structure of 16d-bound SPNS2, likely prohibiting the inhibitor from climbing up to reach the polar “ladder” of the intracellular cavity. In addition, the imidazole ring right after the phenol ring in 16d makes it difficult to flip over the narrow gate region. These structural observations shed light on the inhibition mechanism of the first SPNS2 inhibitor.

While we were preparing our manuscript, a similar study reported the structures of SPNS2 in different states bound with S1P and an inhibitor.^13^ A comparison of our S1P-bound SPNS2 structure with the reported inward-facing state 1 structure shows that the structures are very similar with RMSD of 1.013 Å (Supplementary information, Fig. S8a). Both studies uncover a similar substrate binding and a comparable gating mechanism. A superimposition of our 16d-bound SPNS2 with the reported 16d-bound SPNS2 shows that in our structures the hydrophobic chain inserts much deeper into the substrate binding cavity and the head of the inhibitor also adopts a different orientation (Supplementary information, Fig. S8b). We conducted triplicated MD simulations on our 16d-bound SPNS2. The simulations show that the inhibitor 16d is quite stable in our SPNS2 structure (Supplementary information, Fig. S8c and Video S1).

In summary, our study solves the structure of human SPNS2 in apo and substrate binding states as well as the 16d binding state. Together with the reported SPNS2 structures,^13^ our study provides a framework for understanding the S1P transport mechanism and a rational basis for designing inhibitors targeting SPNS2.

### Supplementary information

Supplementary information

Supplementary Video 1

## Data Availability

All data produced or analyzed in this study are included in the main text or the supplementary materials. The cryo-EM density maps and atomic coordinates have been deposited in the Electron Microscopy Data Bank (EMDB) and Protein Data Bank (PDB) under accession numbers EMD-34103 and 7YUB, respectively, for the S1P-bound SPNS2 nanobody/Fab complex; EMD-34104 and 7YUD, respectively, for the FTY720-P-bound SPNS2 nanobody/Fab complex; EMD-34105 and 7YUF, respectively, for the apo SPNS2 nanobody/Fab complex; EMD-37008 and 8KAE, respectively, for the 16d-bound SPNS2 nanobody/Fab complex.
