# Supplementary material for: Structural basis of Sphingosine-1-phosphate transport via human SPNS2
Source: Cell Res. 2023 Dec 20;34(2):177–80. doi: 10.1038/s41422-023-00913-0 (PMC10837145; doi:10.1038/s41422-023-00913-0)
Supplement: Supplementary file 1 — Supplementary information [file 41422_2023_913_MOESM1_ESM.pdf]

Supplementary Information

Structural basis of Sphingosine-1-phosphate transport via human SPNS2

Yaning Duan[1,7], Nancy C.P. Leong[2,7], Jing Zhao[3,7], Yu Zhang[1], Dat T. Nguyen[2,4,5,6], Hoa T.T. Ha[2], Na Wang[1], Ruixue Xia[1], Zhenmei Xu[1], Zhengxiong Ma[1], Yu Qian[1], Han Yin[1], Xinyan Zhu[1], Anqi Zhang[1], Changyou Guo[1], Yu Xia[3], Long N. Nguyen[2,4,5,6,*], Yuanzheng He[1,*]

[1]Laboratory of Receptor Structure and Signaling, HIT Center for Life Sciences, School of Life Science and Technology, Harbin Institute of Technology, Harbin 150001, China

[2]Department of Biochemistry, Yong Loo Lin School of Medicine, National University of Singapore, 8 Medical Drive, Singapore, 117597, Singapore

[3]MOE Key Laboratory of Bioorganic Phosphorus Chemistry & Chemical Biology, Department of Chemistry, Tsinghua University, Beijing 100084, China

[4]Life Sciences Institute, Immunology Programme, National University of Singapore, Singapore

[5]Immunology Translational Research Program, Yong Loo Lin School of Medicine, National University of Singapore, Singapore 117456.

[6]Singapore Lipidomics Incubator (SLING), Life Sciences Institute, National University of Singapore, Singapore 117456.

[7]These authors contributed equally: YD, NCPL, JZ.

* Corresponding authors: bchnnl@nus.edu.sg (L.N.N) and ajian.he@hit.edu.cn (Y.H.)

Materials and Methods

Constructs

The codon-optimized human SPNS2 (UniProt accession number: Q8IVW8) was subcloned into pFastBac plasmid with a HA-signal peptide sequence on its N-terminus, a tobacco etch virus (TEV) cleavage site and 2 fused maltose binding proteins at its C-terminus. pRH2.2-NabFab was a gift from Dr. Anthony A. Kossiakoff [1]. The Anti-Fab Nb was codon-optimized and cloned into pET24a.

Protein expression and purification

Bacmid preparation and virus production were performed according to the Bac-to-Bac baculovirus system manual (Gibco, Invitrogen). Baculovirus encoding the SPNS2-TEV-2MBP proteins were infected into *Spodoptera frugiperda* (*Sf9*) cells at a density of 2×10^6 cells per ml at a ratio of 1:100 (virus volume versus cell volume). Cells were collected after 48 h shaking at 27 °C at 110 rpm, by centrifugation, washed in ice-cold PBS and stored at −80°C until further use. Cell pellets were thawed on ice and resuspended in 20 mM HEPES (pH 7.5), 150 mM NaCl, and protease inhibitors, and homogenized by a Dounce homogenizer followed by centrifugation (45,000 rpm for 30 mins). The membrane was solubilized with 1% (w/v) lauryl maltose neopentyl glycol (LMNG, Anatrace) and 0.2% (w/v) CHS (Anatrace) at 4 °C for 3 h and ultracentrifuged at 45,000 r.p.m. at 4 °C for 50 min. The supernatant was incubated with an amylose column for 2h and washed with a buffer of 20 mM HEPES (pH 7.5), 150 mM NaCl and 0.01% LMNG/0.002% CHS and eluted with the same buffer plus 10mM maltose. After concentration and TEV cutting

overnight at 4 ℃, the elution was loaded on a Superdex 200 Increase 10/300 GL (Cytiva) gel infiltration column with a buffer of 20 mM HEPES (pH 7.5), 150 mM NaCl and 0.00075% (w/v) LMNG, 0.0002% (w/v) CHS (Anatrace), 0.00025% (w/v) glyco-diosgenin (GDN,Anatrace).Peak fractions were concentrated and stored at −80℃.

Animals

Male alpaca of 1-year old was raised in an open farm with free access to food and water. Animal experiments were carried out in accordance with the Guide for the Care and Use of Laboratory Animals (8th edition) and approved by the Institutional Animal Care and Use Committee of Harbin Institute of Technology (HIT/IACUC).

Nanobody, NabFab and Anti-Fab-Nb generation, expression and purification

Nanobodies that specially bound to SPNS2 were generated according the reported protocol [2,3]. In brief, one alpaca was immunized six times with a total 1 mg of SPNS2 reconstituted in detergent. Five days after the final boost, blood was taken from the alpaca to isolate peripheral blood lymphocytes. RNA was purified from the lymphocytes and reverse transcribed by PCR to obtain cDNA of the open reading frames coding for the nanobodies. The resulting library was cloned into the phage display vector pCANTAB-5E bearing a C-termina E tag and a His_6 tag. Then nanobodies encoded in phages were selected by a wet biopanning process where SPNS2 was hooked on magnetic MBP-tagged beads as bait. SPNS2 specific binding phages were eluted by elute buffer (20mM HEPES, pH 7.5, 150 mM NaCl, 10mM maltose), and after three rounds of selection, periplasmic extracts were

made and analysed using an enzyme-linked immunosorbent assay (ELISA) screens. The sequence of Nb8 in CDR1~CDR4 were grafted to the TC-Nb4 backbone according to the NabFab paper [1], and named as TC-Nb8. Then TC-Nb8 was subcloned into the pET24a vector with a C-termina His_6 tag and expressed in *Escherichia coli* Shuffle T7-K12 at 16 °C. After Ni-NTA (Sigma) affinity purification, nanobodies were further purified by SEC with buffer of 20 mM HEPES pH 7.5, 150 mM NaCl. The purified nanobodies were frozen at −80°C for further use.

NabFab was expressed in *Escherichia coli* C43Pro+ (a gift from Dr. Anthony A. Kossiakoff) at 25°C, purified by Hi Trap Protein L 5ml (Cytiva) affinity chromatography column and further purified by a Resource S (Cytiva) ion exchange column according to the NabFab paper. Anti-Fab Nb was expressed and purified in *Escherichia coli* BL21(DE3) at 16°C according to the report [4].

SPNS2/nanobody/Fab complex assembling

Purified SPNS2 was mixed with TC-Nb8 (3:1 molar ratio) at 4 °C, after 8 hours incubation, the excess TC-Nb8 was removed by centrifugation with a 100 KDa concentration tube at 3,000 rpm for 30 min. NabFab and Anti-Fab Nb were added in the same way step by step and ligands such as S1P and FTY720p were added at 3 μM. Then the complex was loaded on a Superdex 200 Increase 10/300 GL gel infiltration column with a buffer of 20 mM HEPES (pH 7.5), 150 mM NaCl, 0.00075% (w/v) LMNG, 0.0002% (w/v) CHS, 0.00025% (w/v) GDN and ligands. The peak corresponding to the SPNS2/TC-Nb8/NabFab/Anti-Fab

Nb complex was concentrated at about 20 mg/ml and snap frozen for later cryo-EM grid preparation.

Grid preparation and cryo-EM data collection

A 3-5 µl protein complex sample (~20 mg/ml) was loaded to a glow-charged quantifoil R1.2/1.3 Cu holey carbon grids (Quantifoil GmbH), followed with vitrification in liquid ethane on a Vitrobot Mark IV (Thermo Fisher Scientific) instrument at setting of blot force 10, blot time 5 seconds, humidity 100%, temperature 4 ℃. Grids with evenly distributed particles in thin ice were loaded to a FEI 300 kV Titan Krios TEM with a Gatan Quantum energy filter. Images were taken by a Gatan K2 Summit direct electron detector with a super-resolution counting model at pixel size of 0.55 Å (magnitude 64,000 ×). The energy filter slit was set to 20 eV. Each image contains 40 frames with a total exposure time of 7.3 seconds at a dose rate of 1.5 e/Å^2/s (total dose 60 e/Å^2), nominal defocus value varies from -1.2 to -2.2 µm.

Data processing

We use a similar pipeline for data processing as described before [5]. Briefly, 2056-3128 raw movies were binned once (1.1 Å) and motion-corrected by MotionCor2 [6], followed by CTF estimation via CTFFIND 4.1 [7]. About 1.5 million particles were picked by crYOLO [8], followed reference-free 2D classification in RELION [9]. About 644,000 particles of well-defined 2D features were used for initial model generation (cryoSPARC [10] ab initio) and 3D classification. The model was used as reference in RELION 3D classification (~5

classes). The best class (~ 386,000) with clear secondary structure features was selected for a 3D refinement in RELION, followed by a Baysian polishing [11], a 3D refinement and a CTF refinement in RELION. Then subjected to a second round 3D classification (3 classes) with mask on the complex to yield a class of about 288,000 particles for final refinement by the cryoSPARC Non-uniform Refinement, which generated a map of 2.98-3.29 Å, based on the gold standard Fourier Shell Correlation (FSC) = 0.143 criterion. Local resolution estimations were performed using an implemented program in cryoSPARC.

Model building

We used AlphaFold [12] prediction of human SPNS2 (AF-Q8IVW8-v1) was used as initial models for model rebuilding against the electron microscopy map. We used UCSF Chimera [13] to dock models into the electron microscopy density map then subjected to iterative manual adjustment in Coot [14], followed by a rosetta cryoEM refinement [15] and Phenix real space refinement [16]. Structural Figs were prepared in UCSF Chimera, ChimeraX [17] and PyMOL (https://pymol.org/2/).

SPNS2 transport assays

HEK293 cells were seeded onto 12-well plates and maintained in Dulbecco's Modified Eagle's Medium (Gibco) supplemented with 10% fetal bovine serum and 1% penicillin-streptomycin. HEK293 cells were co-transfected with pcDNA3.1 SphK2 and Spns2 plasmids using Lipofectamine 2000 reagent (Thermo Fisher Scientific). After 24 hours post-transfection, cells were washed with DMEM and replenished with DMEM

supplemented with 10mM glycerol disodium phosphate, 5mM sodium fluoride, 1mM semi carbazide and 2.5μM [^{3}H]-sphingosine. The cells were incubated at 37 °C and 5% CO_2 for 3 hours. Cells were washed again with DMEM and replenished with DMEM supplemented with 10mM glycerol disodium phosphate, 5mM sodium fluoride, 1mM semi carbazide, and 0.5% BSA. BSA was used to stimulate S1P release into the medium. The cells were further incubated at 37 °C and 5% CO_2 for 2-3 hours. After 2-3 hours, the medium was collected for S1P isolation using alkaline method. The cells were subsequently washed with ice-cold DMEM supplemented with 0.5% BSA and lysed with RIPA buffer by shaking for 30 minutes. The cell lysates were also collected for S1P isolation. S1P transport activity of the mutants was first normalized to their protein expression levels quantified from Western blot and then expressed as percentage of wild-type protein.

For testing the effect of different pHs and different cations on the Spns2 transport activity, these transport buffers were used: KCl-0mM (140mM NaCl, 20mM HEPES-NaOH pH 7.5, 2mM $CaCl_2$, 1 g/L D-glucose), KCl-140mM (140mM KCl, 20mM HEPES-KOH pH 7.5, 2mM $CaCl_2$, 1 g/L D-glucose), Choline chloride buffer (140mM choline chloride, 20mM HEPES-NaOH pH 7.5, 2mM CaCl2, 1 g/L D-glucose), KCl-15mM (15mM KCl, 125mM NaCl, 20mM HEPES-NaOH pH 7.5, 2mM CaCl2, 1 g/L D-glucose), KCl-50mM (50mM KCl, 90mM NaCl, 20mM HEPES-NaOH pH 7.5, 2mM CaCl2, 1 g/L D-glucose), KCl-100mM (100mM KCl, 40mM NaCl, 20mM HEPES-NaOH pH 7.5, 2mM CaCl2, 1 g/L D-glucose), pH8.5 buffer: (140mM NaCl, 20mM Tris-HCl pH 8.5, 2mM $CaCl_2$, 1 g/L D-glucose), pH6.5 buffer: (140mM NaCl, 20mM MES pH 6.5, 2mM $CaCl_2$, 1 g/L D-glucose), or Hank's balanced salt solution with 5 mM HEPES pH 7.5 was used.

S1P Isolation and Liquid Scintillation Counting.

An amount of 400μL of the medium was collected and added with 260μL of methanol, followed by 800μL of chloroform: methanol (3:1 v/v) and 30μL of 25% ammonium hydroxide and 300μL of 150mM potassium chloride. The mixture was then vortexed for 10 minutes and centrifuged at max 15k RPM for 5 minutes at room temperature. An amount of 800μL of the upper aqueous phase was collected into scintillation vials containing 4mL of EcoLite scintillation cocktail (MP Biomedicals). The samples were counted on TriCarb Liquid Scintillation Counter (Perkin Elmer). Radioactive signals were expressed as DPM (disintegration per minute).

LC-MS analysis of S1P and FTY720p in the purified SPNS2 complex

100 μL sample solution (5 mg/mL protein complex) was added in a 2 mL-vial, with 1365 μL ice-cold MTBE/MeOH/2 M HCl (200:60:13, *v/v/v*), vortex for 1 min. Then, 250 μL 0.1N HCl was added, vortex for 5 min. After 5 min centrifugation, the upper solution was obtained, dried under nitrogen flow, and redissolved in 90 μL MeOH. The solution was then added with 7.5 μL TMSD solution (1.5 mM) and vortexed at 25°C for 20 min. The methylation was terminated by adding 2.5 μL acetic acid. The obtained solution was then subjected to HPLC(HILIC column)-MS analysis. The detection was under positive mode.

Proteoliposome transport assay.

The method for preparation of proteoliposomes was described previously [18]. Briefly, liposomes were generated using 80 percent molar of POPC and 20 percent molar of

cholesterol in buffer containing 20mM HEPES and 150mM NaCl at pH 7.5 (buffer A). Liposomes were loaded with 0.1% fatty acid free BSA. To prepare SPNS2 proteoliposomes, 50 μg SPNS2 purified protein was reconstituted in 160 μl of liposomes in the presence of 0.11% Triton-X100. Excess detergents were then removed with SM2 Bio-beads (Bio-rad). The proteoliposomes were washed 2 times with buffer A before resuspended in 320 μl buffer A. Liposomes loaded with 0.1% BSA were used as control (control liposomes). For S1P transport assay, SPNS2 proteoliposomes and control liposomes were incubated with 2.5 μM [^{3}H]-S1P or 2.5-5 μM NBD-S1P for 1hr at 37 ℃. The transport assay was stopped by adding 1ml cold buffer A. The excessive ligand was removed by washing the proteoliposomes in cold buffer A three times. For transport assays with [^{3}H]-S1P, radioactive signals were quantified by scintillation counter (Tricarb-4810TR; PerkinElmer). For transport with NBD-S1P, the NBD-S1P was extracted from the proteoliposomes and spotted on TLC plate for quantification using fluorescent scanner (ChemiDoc MP; Bio-rad).

Molecular dynamics simulation

For SPNS2, the atomic coordinates of apo, S1P-bound, FTY720p-bound SPNS2 and 16d-bound in the near-end refinement were used as initial models in the MD simulation, the missing intracellular loop (285-300) was filled with loop from AlphaFold prediction of SPNS2 (AF-Q8IVW8-v1). All models are prepared and parameterizes in CHARMM-GUI [18,19]. Protonation states of all titratable residues were assigned at pH 7.0. Histidine was modelled as neutral. The transporters were inserted into a bilayer lipid contain POPC (palmitoyl-2-oleoyl-sn-glycero-3-phosphocholine) and cholesterol at ratio of 4:1, the

membrane size is 65 x 65 Å with 22.5 Å water in the top and bottom (final system dimensions ~65x65x120 Å). Ion was set to either 0.15 M KCl. The Amber force fields were set to: protein FF19SB, lipid LIPID17, water TIP3P and ligand GAFF2. The simulations were performed by Amber20 package [20]. The system was first energy minimized for solvent and all atoms, heat to 300 K in 300 ps and then equilibrated for 700 ps, followed by three independent production runs of 200 ns with a time step of 2 fs. During simulations, Particle mesh Ewald algorithm were applied for the calculation of long-range electrostatic interaction and a cutoff of 10 Å were applied for short-range electrostatic interaction and van der Waals interactions. All bonds with hydrogens are constrained by SHAKE algorithm. The system temperature (300 K) and pressure (1 atm) were controlled by Langevin thermostat and Berendsen barostat, respectively. The trajectories were analyzed and visualized in VMD [21]. Videos were recorded by VMD.

Western Blot analysis

HEK293 cells were seeded onto 12-well plates and maintained in Dulbecco's Modified Eagle's Medium (Gibco) supplemented with 10% fetal bovine serum and 1% penicillin-streptomycin. HEK293 cells were transfected with Lipofectamine 2000 reagent (Thermo Fisher Scientific). 24 hours post-transfection, cells were washed with PBS once and whole cells lysates were prepared with RIPA buffer supplemented with protease inhibitor cocktail (Roche). The same amount of protein lysates were loaded for SDS PAGE and transferred to nitrocellulose membrane. The membranes were blocked for 1 hour at room temperature with 5% milk in TBS with 0.1% Tween 20 (TBST). SPNS2 antibody was diluted at 1:1000 in the blocking buffer and incubated with membranes for 90 minutes at room temperature

or overnight at 4 °C. Membranes were then washed with TBST three times for 5 minutes each time. Secondary antibodies were diluted in the blocking buffer and incubated with membranes for 1 hour at room temperature. Washing steps were repeated. The membranes were imaged using the ChemiDoc MP imaging system (Bio-rad). The antibodies used included in-house rabbit anti-Spns2 (1:1000), mouse anti-GAPDH (1:4000), donkey anti-rabbit IR800 (1:10000) and goat anti-mouse IR680 (1:10000).

Immunofluorescent Staining

HEK293 cells were seeded onto 24-well plates with coverslips and maintained in Dulbecco's Modified Eagle's Medium (Gibco) supplemented with 10% fetal bovine serum and 1% penicillin-streptomycin. HEK293 cells were transfected with Lipofectamine 2000 reagent (Thermo Fisher Scientific). The cells were washed with PBS twice and fixed in 4% PFA for 15 minutes at room temperature, followed by washing with PBS twice, and permeabilized in PBST (PBS with 0.5% triton-X) for 15 minutes at room temperature. The cells were subsequently washed with PBS and blocked in 5% normal goat serum for one hour before staining with SPNS2 antibody at 1:250 dilutions for 1hr. The cells were counter-stained with DAPI and imaged with laser confocal microscope.

Supplementary video information

Supplementary Video S1. Movie track of MD simulation of 16d-bound SPNS2. The length is 200ns, interval is 5 steps.

References

1 Bloch, J. S. *et al.* Development of a universal nanobody-binding Fab module for fiducial-assisted cryo-EM studies of membrane proteins. *Proc Natl Acad Sci U S A* **118**, doi:10.1073/pnas.2115435118 (2021).

2 Pardon, E. *et al.* A general protocol for the generation of Nanobodies for structural biology. *Nature protocols* **9**, 674-693, doi:10.1038/nprot.2014.039 (2014).

3 Vincke, C. *et al.* Generation of single domain antibody fragments derived from camelids and generation of manifold constructs. *Methods Mol Biol* **907**, 145-176, doi:10.1007/978-1-61779-974-7_8 (2012).

4 Tsutsumi, N. *et al.* Structure of human Frizzled5 by fiducial-assisted cryo-EM supports a heterodimeric mechanism of canonical Wnt signaling. *eLife* **9**, doi:10.7554/eLife.58464 (2020).

5 Wang, N. *et al.* Structural basis of leukotriene B4 receptor 1 activation. *Nature communications* **13**, 1156, doi:10.1038/s41467-022-28820-9 (2022).

6 Zheng, S. Q. *et al.* MotionCor2: anisotropic correction of beam-induced motion for improved cryo-electron microscopy. *Nature methods* **14**, 331-332, doi:10.1038/nmeth.4193 (2017).

7 Rohou, A. & Grigorieff, N. CTFFIND4: Fast and accurate defocus estimation from electron micrographs. *Journal of structural biology* **192**, 216-221, doi:10.1016/j.jsb.2015.08.008 (2015).

8 Wagner, T. *et al.* SPHIRE-crYOLO is a fast and accurate fully automated particle picker for cryo-EM. *Commun Biol* **2**, 218, doi:10.1038/s42003-019-0437-z (2019).

9 Fernandez-Leiro, R. & Scheres, S. H. W. A pipeline approach to single-particle processing in RELION. *Acta Crystallogr D Struct Biol* **73**, 496-502, doi:10.1107/S2059798316019276 (2017).

10 Punjani, A., Rubinstein, J. L., Fleet, D. J. & Brubaker, M. A. cryoSPARC: algorithms for rapid unsupervised cryo-EM structure determination. *Nature methods* **14**, 290-296, doi:10.1038/nmeth.4169 (2017).

11 Zivanov, J., Nakane, T. & Scheres, S. H. W. A Bayesian approach to beam-induced motion correction in cryo-EM single-particle analysis. *IUCrJ* **6**, 5-17, doi:10.1107/S205225251801463X (2019).

12 Jumper, J. *et al.* Highly accurate protein structure prediction with AlphaFold. *Nature* **596**, 583-589, doi:10.1038/s41586-021-03819-2 (2021).

13 Pettersen, E. F. *et al.* UCSF Chimera--a visualization system for exploratory research and analysis. *J Comput Chem* **25**, 1605-1612, doi:10.1002/jcc.20084 (2004).

14 Emsley, P. & Cowtan, K. Coot: model-building tools for molecular graphics. *Acta Crystallogr D Biol Crystallogr* **60**, 2126-2132, doi:10.1107/S0907444904019158 (2004).

15 Wang, R. Y. *et al.* Automated structure refinement of macromolecular assemblies from cryo-EM maps using Rosetta. *eLife* **5**, doi:10.7554/eLife.17219 (2016).

16 Adams, P. D. *et al.* PHENIX: a comprehensive Python-based system for macromolecular structure solution. *Acta Crystallogr D Biol Crystallogr* **66**, 213-221, doi:10.1107/S0907444909052925 (2010).
17 Pettersen, E. F. *et al.* UCSF ChimeraX: Structure visualization for researchers, educators, and developers. *Protein science : a publication of the Protein Society* **30**, 70-82, doi:10.1002/pro.3943 (2021).
18 Jo, S., Kim, T., Iyer, V. G. & Im, W. CHARMM-GUI: a web-based graphical user interface for CHARMM. *J Comput Chem* **29**, 1859-1865, doi:10.1002/jcc.20945 (2008).
19 Lee, J. *et al.* CHARMM-GUI supports the Amber force fields. *The Journal of chemical physics* **153**, 035103, doi:10.1063/5.0012280 (2020).
20 Case, D. A. *et al.* The Amber biomolecular simulation programs. *J Comput Chem* **26**, 1668-1688, doi:10.1002/jcc.20290 (2005).
21 Humphrey, W., Dalke, A. & Schulten, K. VMD: visual molecular dynamics. *J Mol Graph* **14**, 33-38, 27-38, doi:10.1016/0263-7855(96)00018-5 (1996).

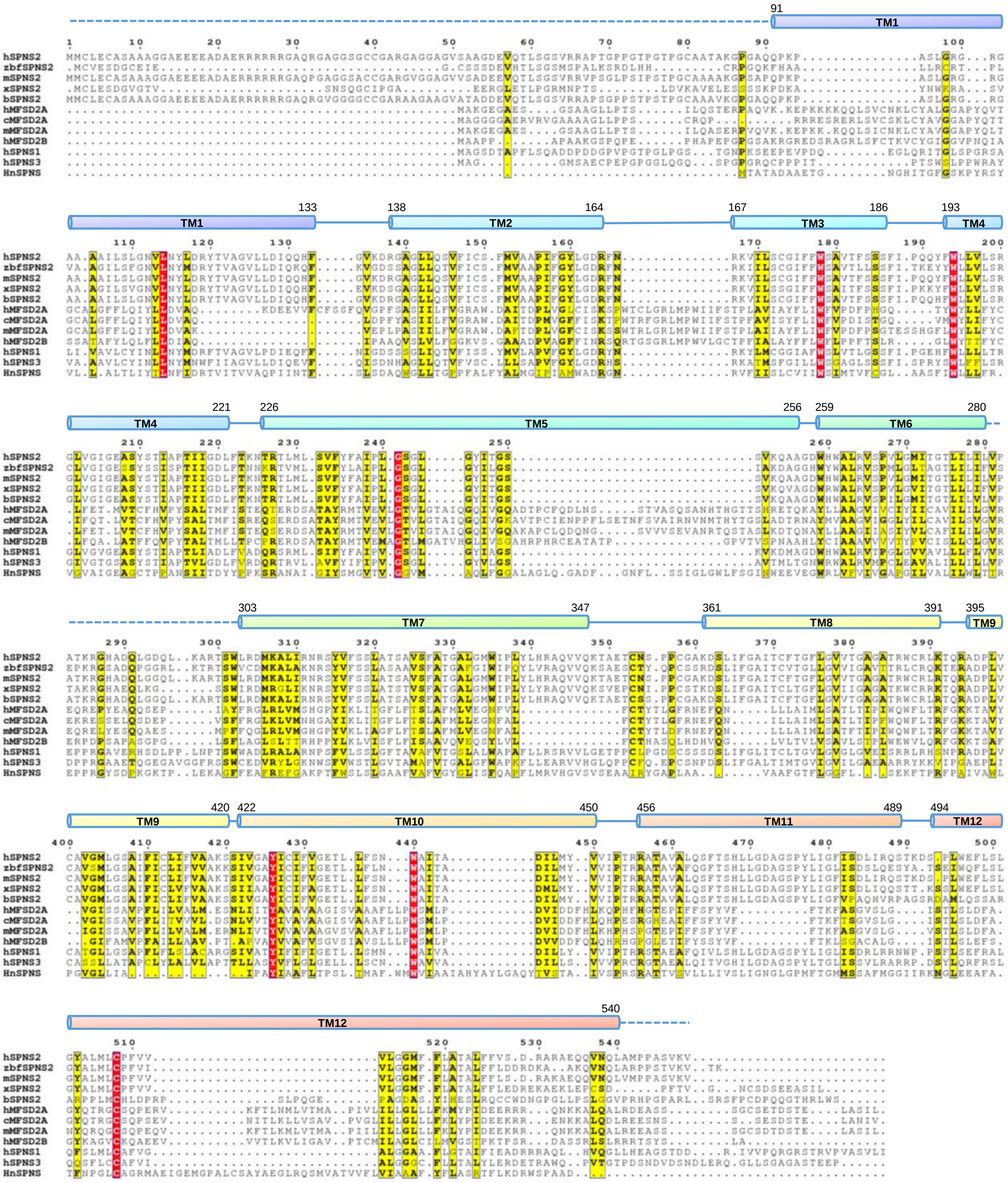

Supplementary information, Fig. S1 Sequence alignment of SPNS, MFSD2A and their homologues. *Homo sapiens* (h), *Zebra fish* (zbf), *Mus musculus* (m), *Xenopus laevis* (x), *Bos Taurus* (b), *Chicken* (c) and *Hyphomonas neptunium(Hn)*.

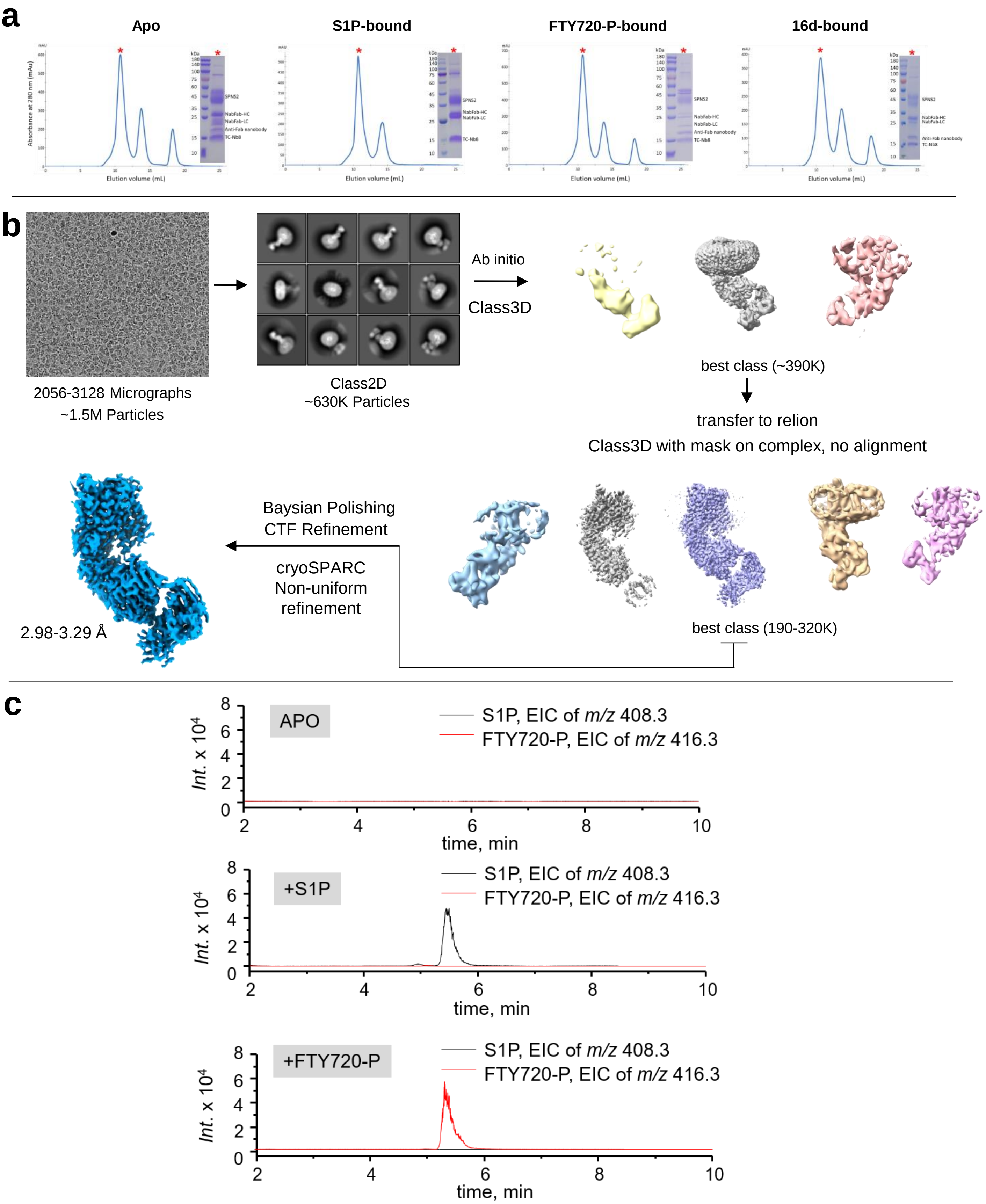

Supplementary information, Fig. S2 Purification and cryo-EM analysis of SPNS2/nanobody/Fab complex. a, Size-exclusion chromatography profile of SPNS2/nanobody/Fab complexes. **b,** Flow-chart of cryo-EM data process of SPNS2. **c,** LC-MS analysis of S1P, FTY720-P in purified SPNS2 protein.

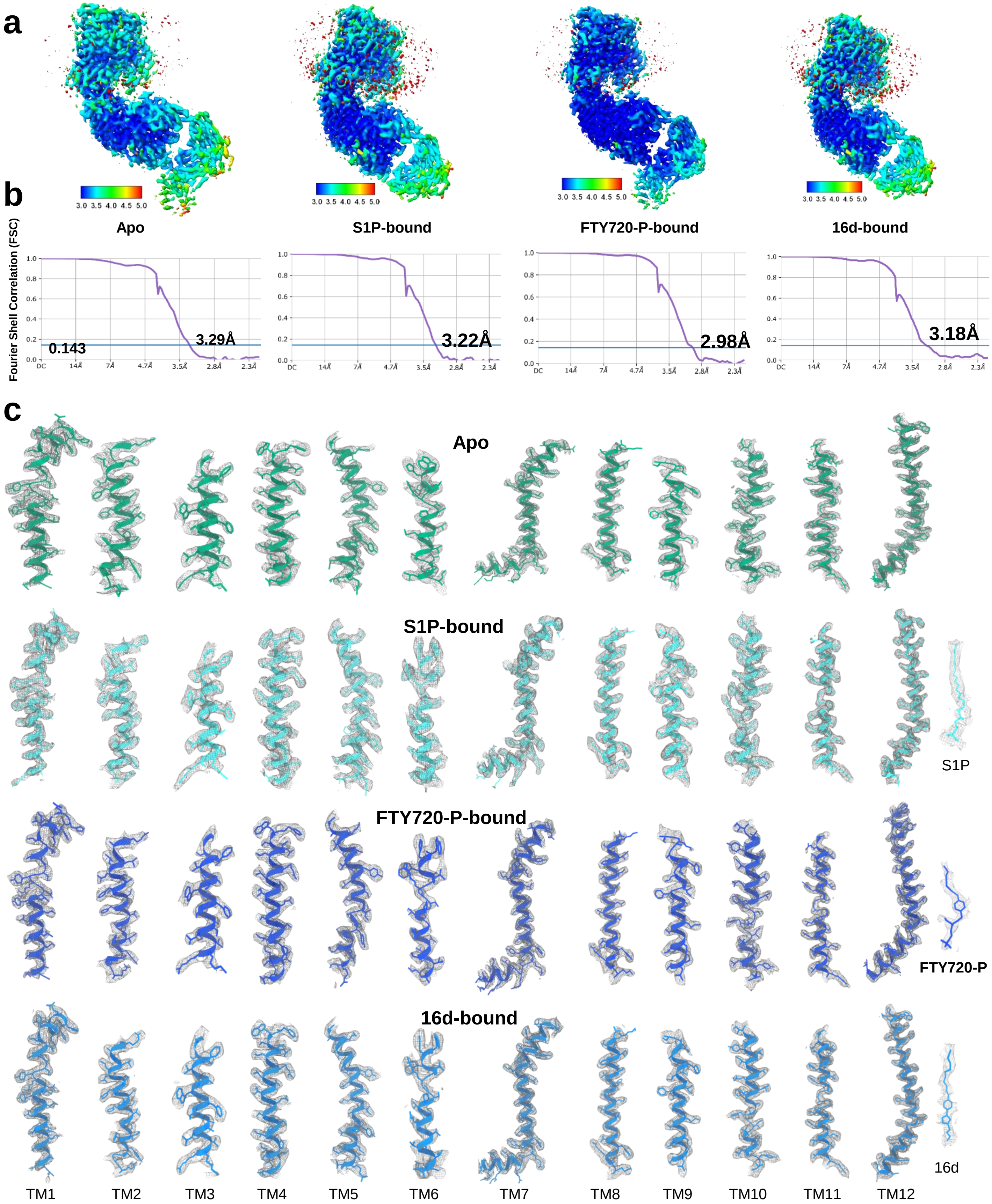

Supplementary information, Fig. S3 Resolution and representative map of SPNS2 complex. a, Local resolution analysis of SPNS2/nanobody/Fab complexes. **b,** FSC curves of SPNS2/nanobody/Fab complexes, the resolution was assessed by the Gold Standard of FSC=0.143. **c,** Cryo-EM density map of representative regions SPNS2. Map level was set to 0.8 in chimeraX.

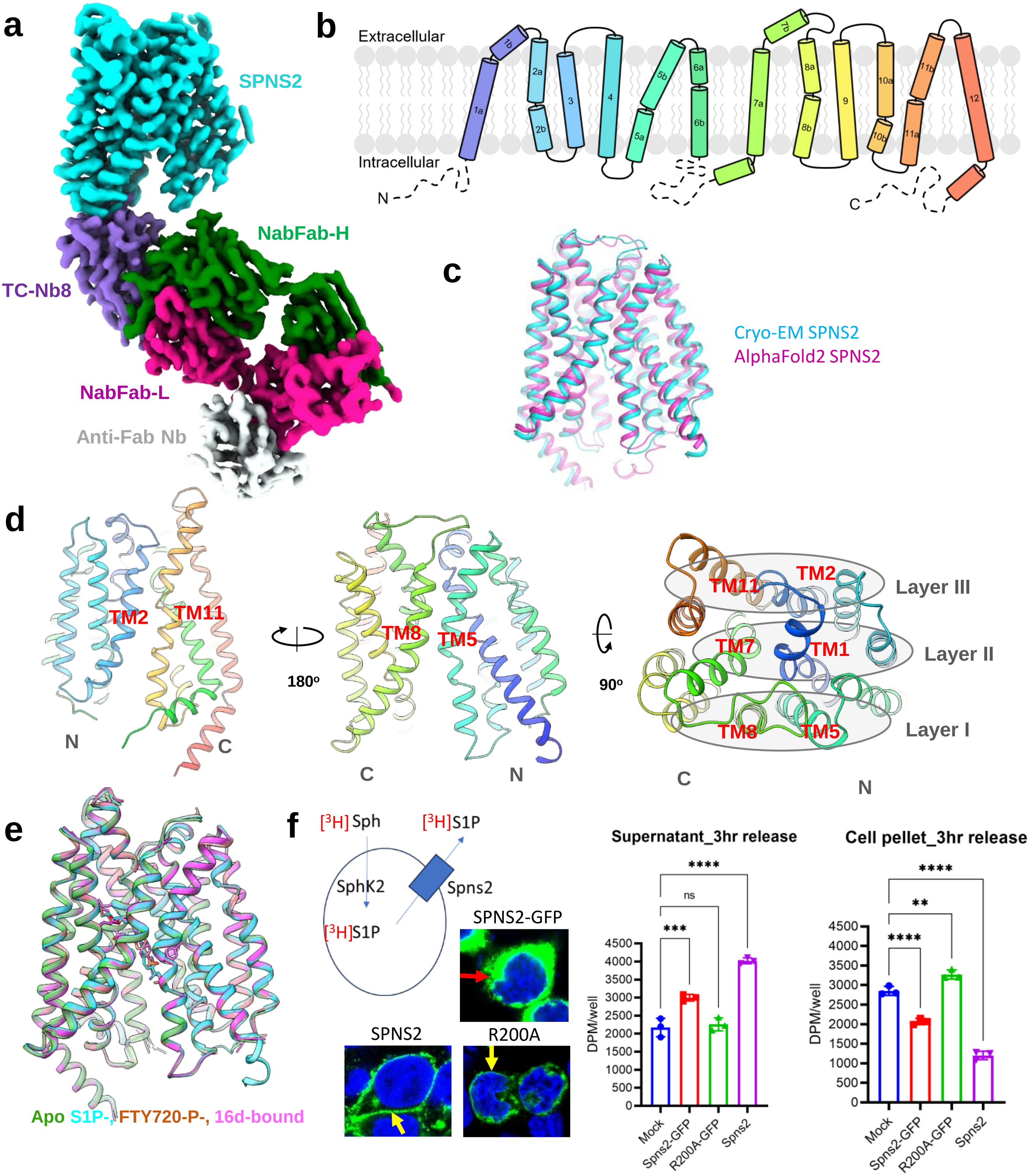

Supplementary information, Fig.S4 Additional information of the overall structure of SPNS2. a, The overall map of SPNS2/nanobody/NabFab/anti-Fab Nb complex. **b,** Topology of SPNS2. Disordered regions are shown as dashed lines. **c**, A comparison of the S1P-bound SPNS2 with the AlphaFold prediction of SPNS2. **d**, The N domain and C domain are held together on the extracellular side by three layers of helix interaction. **e,** An superimposition of apo, S1P-,FTY720-P- and 16d-bound SPNS2. **f,** The establishment of a transport assay for SPNS2. Note that GFP-tagged SPNS2 exhibited reduced S1P transport activity compared to untagged SPNS2. It is due to its defective localization on the plasma membrane (red arrow). Thus, we used untagged SPNS2 for the mutagenesis study for the key amino acids. Data are presented as mean values ± SD; *n* = 3 independent samples; n.s., no significance; *, $p < 0.05$; **, $p < 0.01$; ***, $p < 0.001$, ****, $p<0.0001$. One-way and Two-way ANOVA were used.

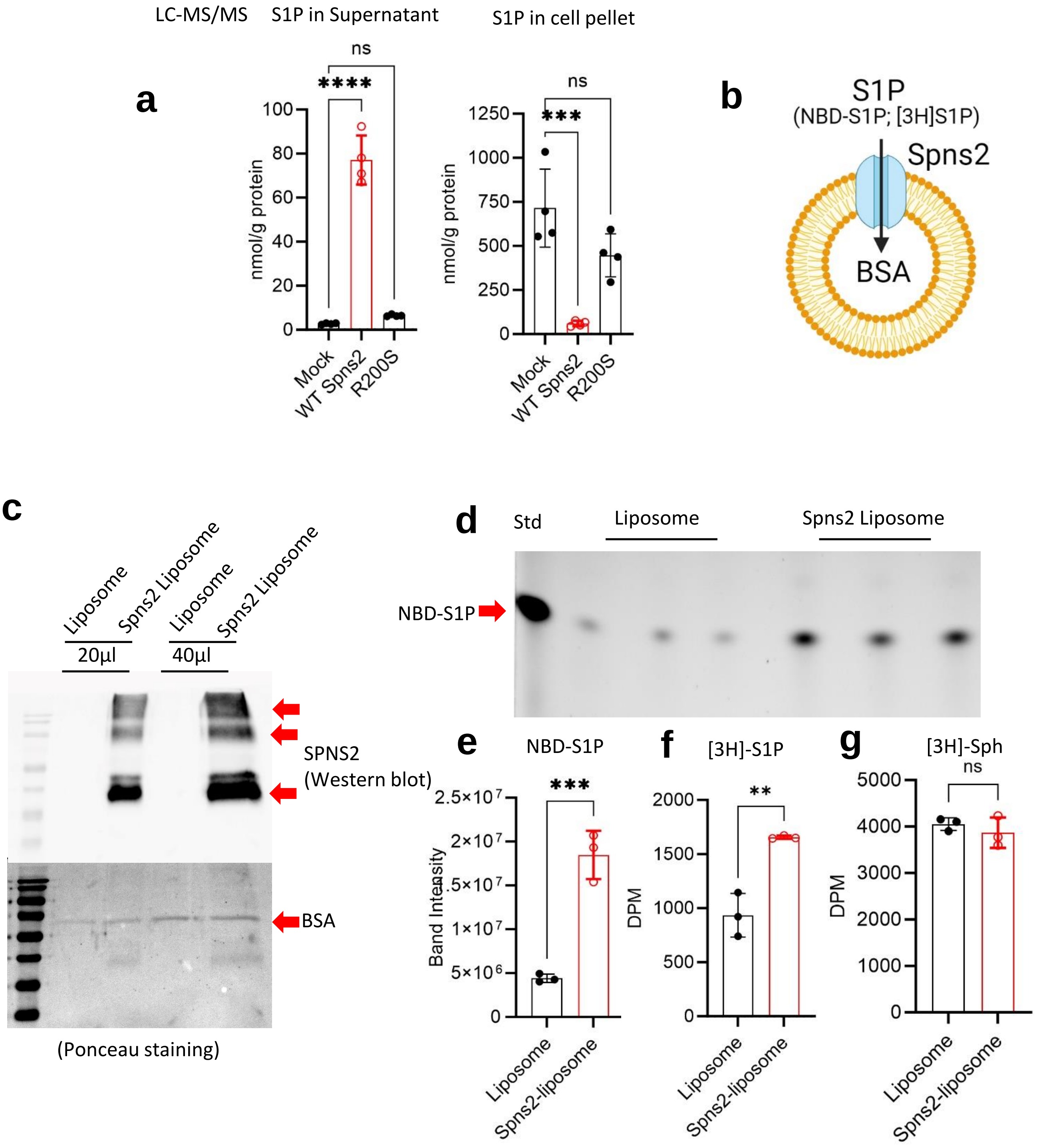

Supplementary information, Fig.S5 In vitro transport assay of SPNS2. a, Mass spectrometry deletion of S1P release into the supernatant after sphingosine was used as the substrate. The results validate that the radioactive signals in the supernatant in our transport assays was S1P, not other metabolites from sphingosine. **b-g,** S1P transport assays using proteoliposomes. WT SPNS2 protein was reconstituted in liposomes. Control liposomes and SPNS2 liposomes were loaded with 0.1% BSA. 2.5 µM NBD-S1P (in **d-e**) or [3H]-S1P (in **f**) was used as ligands for the proteoliposome assays. SPNS2 exhibited direct transport of S1P ligands, but not sphingosine (2.5 µM Sph in **g**). Data are presented as mean values ± SD; *n* = 3 independent samples; n.s., no significance; *, $p < 0.05$; **, $p < 0.01$; ***, $p < 0.001$, ****, p<0.0001. One-way and Two-way ANOVA were used.

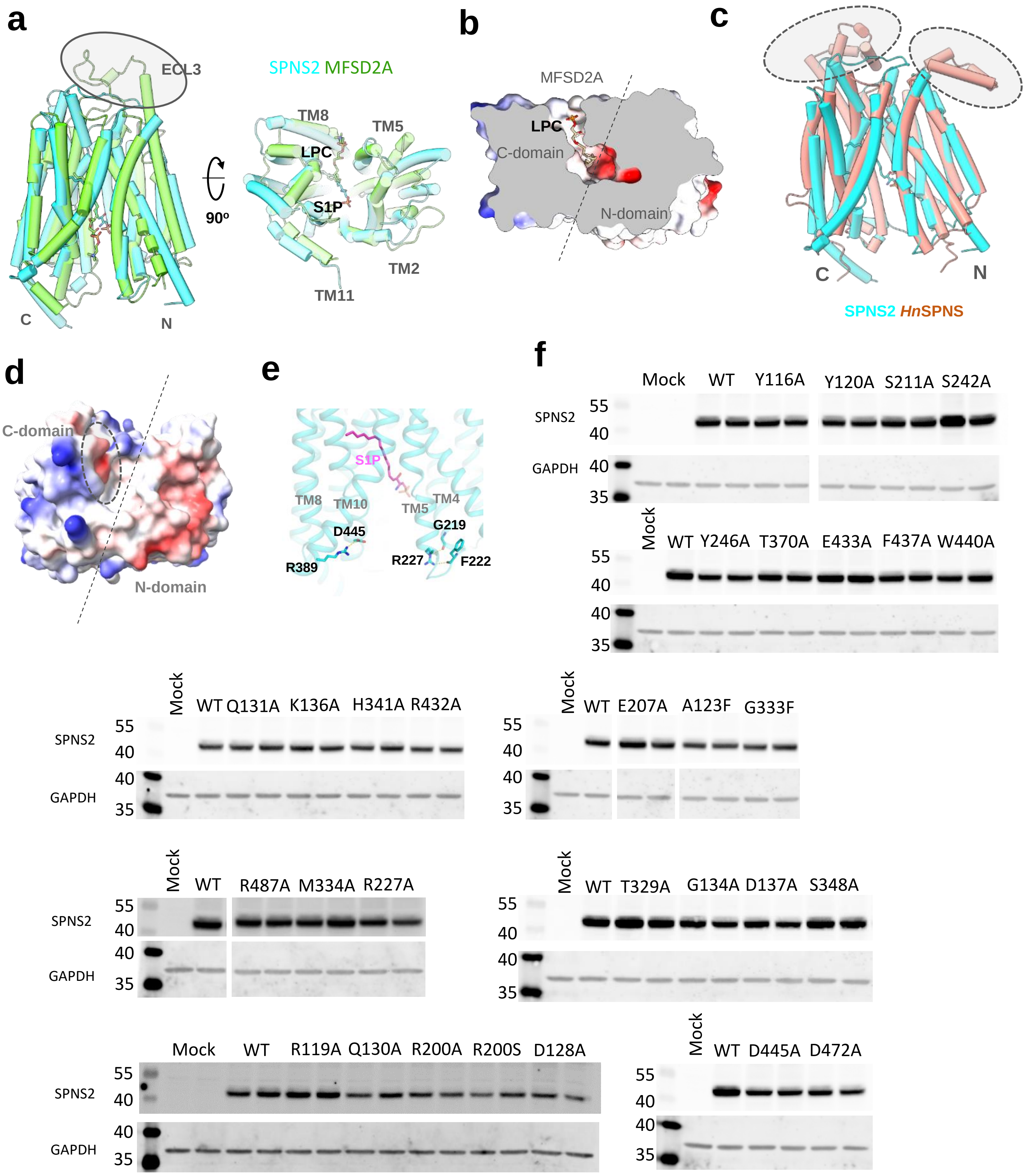

Supplementary information, Fig. S6 Comparisons of SPNS2 with MFSD2A and *Hn*SPNS and the proteins expression level data of SPNS2. a, A comparison of SPNS2 with the inward-facing chicken MFSD2A (PDB: 7mjs). **b,** An electrostatics analysis of the chicken inward-facing MFSD2A from the bottom (intracellular side) view. **c,** A comparison of human SPNS2 with the bacterial *Hn*SPNS (PDB: 6e8j). **d**, The surface electrostatics of SPNS2 on the extracellular side. **e**, The intracellular entry of SPNS2 at the TM5/TM8 side. **f,** Western-Blot of SPNS2 mutants.

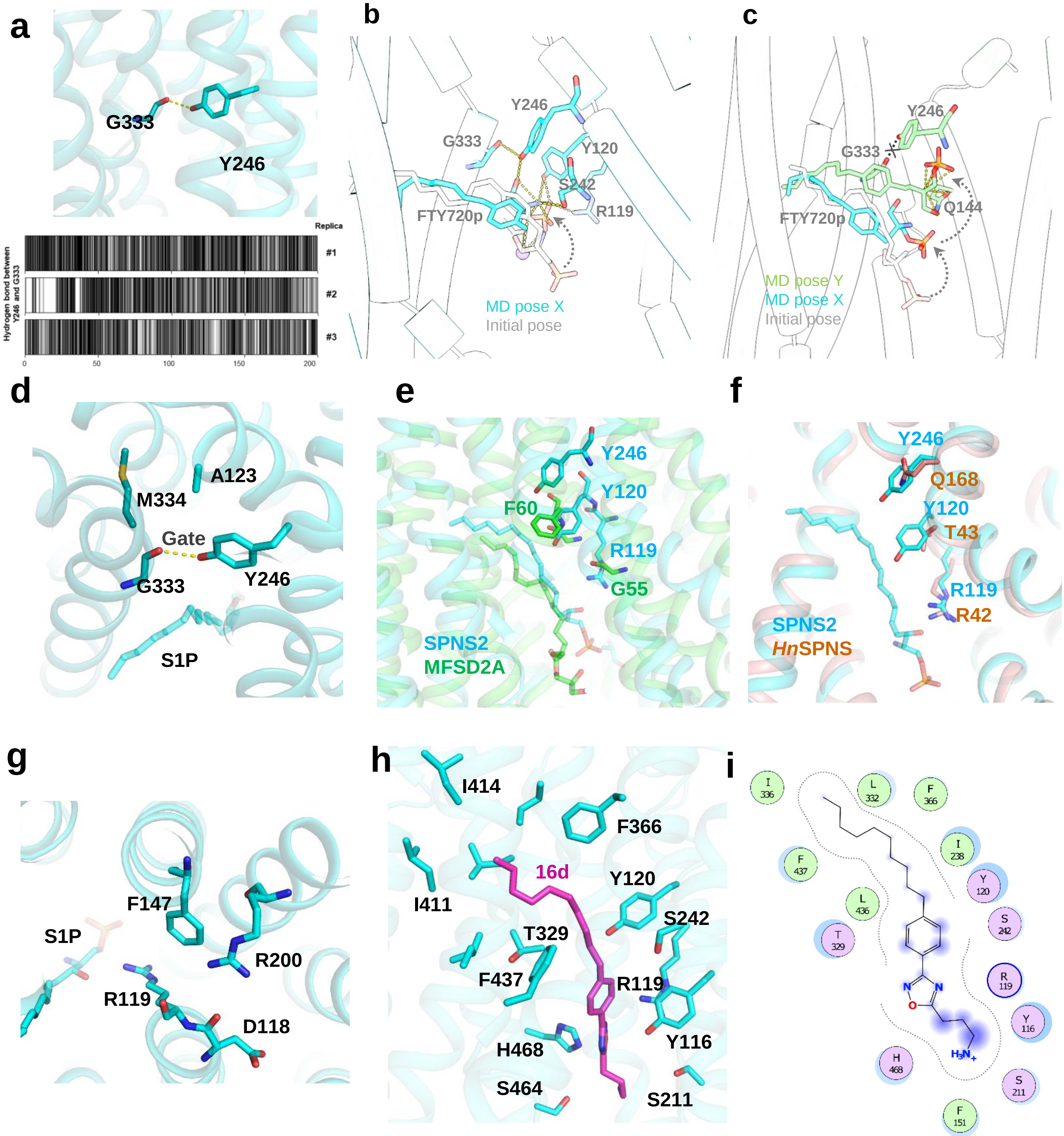

Supplementary information, Fig. S7 Additional information of the gate area and inhibitor-bound SPNS2. a, MD analysis of the Y246/G333 interaction. Upper panel, a snap-shot of the Y246/G333 interaction; lower panel, statistics of hydrogen bond interaction of Y246/G333 in MD simulations of apo SPNS2, the cutoff of hydrogen bond is set to 3.0 Å with 20 °tolerance. **b-c**, Snap-shots of MD simulations on FTY720-P-bound SPNS2 in a 1 µs run. **d**, Hydrophobic residues M334 and A123 sits above the gate. **e**, A structural comparison of the "ladder" area between SPNS2 and chicken MFSD2A (PDB: 7mjs). **f,** A structural comparison of the "ladder" area between SPNS2 and *Hn*SPNS (PDB: 6e8j). **g,** A detailed analysis of R200 in the gating area of SPNS2. **h**, **i**, The position and interaction map of 16d in the intracellular cavity of SPNS2.

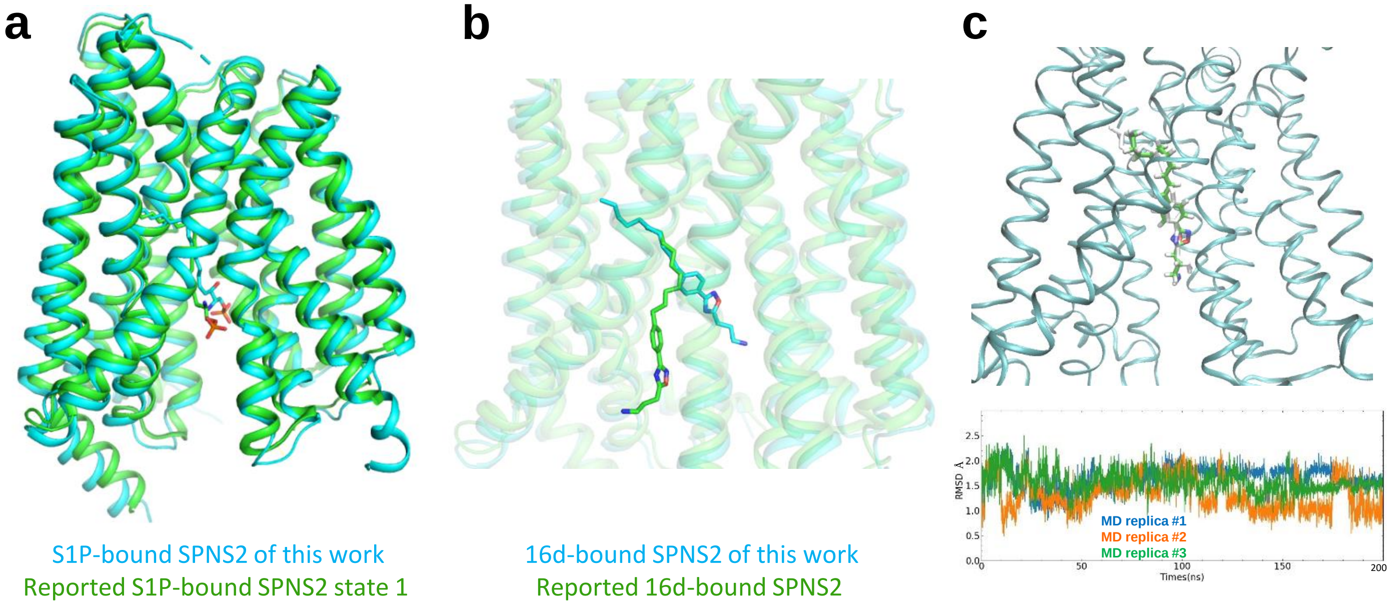

Supplementary information, Fig. S8 A comparison of SPNS2 of this study with the reported study from the Lee group. a, A comparison of S1P-bound SPNS2 with the reported S1P-bound SPNS2 in state 1 (PDB: 8ex4). **b,** A structural comparison of the 16d-bound SPNS2 with the reported 16d-bound SPNS2 (PDB: 8g92). **c,** A snap-shot of the MD simulations of 16d-bound SPNS2 in a 200 ns run.

Supplementary information, Table S1 Cryo-EM data collection and refinement statistics

	SPNS2/S1P/TC-Nb8/NabFab<br>EMD-34103<br>7YUB	SPNS2/FTY720-P/TC-Nb8/NabFab/Anti-Fab Nb<br>EMD-34104<br>7YUD	SPNS2/apo/TC-Nb8/NabFab/Anti-Fab Nb<br>EMD-34105<br>7YUF	SPNS2/16d/TC-Nb8/NabFab/Anti-Fab Nb<br>EMD-37008<br>8KAE
**Data collection and processing**				
Magnification	130,000	130,000	130,000	130,000
Voltage (kV)	300	300	300	300
Electron exposure ($e^-/Å^2$)	60	60	60	60
Defocus range (μm)	1.2-2.2	1.2-2.2	1.2-2.2	1.2-2.2
Pixel size (Å)	0.55	0.55	0.55	0.55
Symmetry imposed	C1	C1	C1	C1
Initial particle image (no.)	1.82M	1.43M	1.55M	2.3M
Final particle image (no.)	290k	362k	196k	90.3k
Map resolution (Å)	3.22	2.98	3.29	3.18
FSC threshold	0.143	0.143	0.143	0.143
**Refinement**				
Initial model used (PDB code)	AlphaFold-Q8IVW8-v1	AlphaFold-Q8IVW8-v1	AlphaFold-Q8IVW8-v1	AlphaFold-Q8IVW8-v1
Model Resolution (Å)	NA	NA	NA	NA
Map sharpening *B* factor ($Å^2$)	-144.9	-127.4	-139.9	-107.7
Model composition				
Non-hydrogen atoms	15283	8501	8475	8440
Protein residues	1006	1114	1114	1105
Ligands	1	1	0	0
B factor ($Å^2$)				
Protein	61.42	61.91	77.37	110.61
Ligand	61.97	61.66		74.09
R.m.s. deviations				
Bond length (Å)	0.005	0.008	0.007	0.013
Bond angles (°)	1.042	1.209	1.105	1.042
Validation				
MolProbity score	1.15	1.27	1.17	1.84
Clashscore	1.77	1.78	1.67	6.93
Poor rotamers (%)	0	0	0	0
Ramachandran plot				
Favored (%)	96.59	95.19	96.28	92.76
Allowed (%)	3.41	4.81	3.72	7.24
Disallowed	0	0	0	0
